# Sequence variations of *GRM6* in patients with high myopia

**Published:** 2009-10-19

**Authors:** Xiaoyu Xu, Shiqiang Li, Xueshan Xiao, Panfeng Wang, Xiangming Guo, Qingjiong Zhang

**Affiliations:** State Key Laboratory of Ophthalmology, Zhongshan Ophthalmic Center, Sun Yat-sen University, Guangzhou, China

## Abstract

**Purpose:**

Mutations in the glutamate receptor metabotropic 6 gene (*GRM6*) have been identified in patients with congenital stationary night blindness (CSNB1B). High myopia is usually observed in CSNB1B patients. This study tested if any mutations in *GRM6* were solely responsible for high myopia.

**Methods:**

DNA was prepared from the venous leukocytes of 96 Chinese patients with high myopia (refraction of spherical equivalent of at least −6.00 diopters [D]) and 96 controls (refraction of spherical equivalent between −0.50 D and +2.00 D with normal visual acuity). The coding regions and adjacent intronic sequence of *GRM6* were amplified by a polymerase chain reaction (PCR) and then analyzed by cycle sequencing. Detected variations were evaluated in normal controls by heteroduplex-single-strand-conformation (SSCP) polymorphism analysis or restriction fragment polymorphism (RFLP).

**Results:**

Four novel variations predicted to have potential functional changes were identified: c.67-82delCAGGCGGGCCTGGCGCinsT (p.Gln23_Arg28delinsCys), c.858-5a>g (r.spl?), c.1172G>A (p.Arg391Gln), and c.1537G>A (p.Val513Met). Except for c.1172G>A, the other three were not detected in the 96 controls. In addition, five rare variations—(c.72G>A, c.504+10g>t, c.726-50g>c, c.1359C>T, and c.1383C>T)—and one common variation (c.2437-6g>a) without predicted functional consequences and nine known single nucleotide polymorphisms (SNPs) were also detected.

**Conclusion:**

Three novel variations with potential functional consequences were identified in the *GRM6* of patients with high myopia, suggesting a potential role in the development of myopia in rare cases.

## Introduction

The glutamate receptor metabotropic 6 gene (*GRM6*; OMIM 604096), mapped to 5q35, contained 10 exons and encoded an 877 amino acid protein, mGluR6. As a member of Group III mGluRs (mGluR4, 6, 7, and 8), it contains a signal peptide, a large bi-lobed extracellular NH_2_-terminal domain containing the glutamate binding site, seven G protein-coupled receptor (GPCR) transmembrane domains and an intracellular COOH-terminal domain [1-3]. In the rat retina, mGluR6 is specifically expressed in ON bipolar cells at the postsynaptic site [4]. In the retinal neural network, an increase in light reduced the release of glutamate from cones and rods, while a decrease in light increases its release, which acted as inputs from photoreceptors to bipolar cells. The visual signals of light and dark were segregated into parallel ON and OFF pathways through ON and OFF bipolar cells. The ON bipolar cells utilize a metabotropic glutamate receptor (mGluR6) for a light-activated depolarization, whereas the OFF bipolar cells utilized ionotropic glutamate receptors (iGluRs) for a light-activated hyperpolarization. ON and OFF bipolar cells made synaptic contacts with ON and OFF ganglion cells and transmitted visual signals to the brain [5-8].

Functional defects involving retinal ON-pathways have been demonstrated by retinal electrophysiology studies in the complete form of congenital stationary night blindness, including CSNB1A (OMIM 310500) and CSNB1B (OMIM 257270) [7,9-12]. Mutations in *NYX* or *GRM6* are responsible for CSNB1A or CSNB1B, respectively [10,11,13-15]. Besides night blindness, high myopia is also frequently documented as a typical sign in CSNB1A patients with *NYX* mutations [13,14,16-18]. A mouse model with *Nyx* mutation and retinal ON-pathway defect has high susceptibility to experimental myopia [19]. Recently, mutations in *NYX* have been reported to associate with high myopia alone without night blindness [20]. Similarly, moderate to high myopia is also a common sign in CSNB1B patients with *GRM6* mutations [10,21]. A mouse model lacking the *GRM6* gene showed a loss of ON response, but an unchanged OFF response to light, demonstrating its essential role in ON synaptic transmission [4].

Congenital stationary night blindness (CSNB) can be caused by mutations in genes *GNAT1, PDE6B, RHO, CABP4, GRK1, GRM6, RDH5, SAG, CACNA1F,* and *NYX* (RetNet). Myopia is not always associated with CSNB, except in cases resulting from mutations in *NYX* and *GRM6* [10,16], suggesting a gene-specific phenotype rather than association with night blindness. As mutations in *GRM6* resulted in phenotypes extremely similar to those in *NYX*, this suggested that *GRM6* might be a candidate susceptibility gene for isolated high myopia. In this study, we analyzed the genomic sequence of *GRM6* in 96 Chinese patients with high myopia.

## Methods

### Subjects

Ninety-six unrelated probands with high myopia and 96 unrelated normal controls were collected from Zhongshan Ophthalmic Center. Informed consent conforming to the tenets of the Declaration of Helsinki was obtained from the participants prior to the study. This study was approved by the Institutional Review Boards of Zhongshan Ophthalmic Center. Ophthalmological examinations were performed by ophthalmologists (Drs. Q.Z. and X.G.). The diagnostic criteria for high myopia were as we previously described [22]: 1) bilateral refraction of –6.00D or lower (spherical equivalent) and 2) no other known ocular or systemic diseases associated with high myopia. Normal controls met the following criteria: 1) bilateral refraction between –0.50 D and +2.00 D with normal visual acuity, 2) no family history of high myopia, and 3) exclusion of known ocular or systemic diseases. The refractive error of all eyes was measured with cycloplegic autorefraction after mydriasis (Mydrin^®^-P, a compound tropicamide; Santen Pharmaceutical Co. Ltd., Osaka, Japan). Genomic DNA was prepared from venous blood.

### Variation analysis

Seven pairs of primers (Table 1) were used to amplify the 10 coding exons and the adjacent intronic sequence of *GRM6* (human genome build 36.2 NC_000005.8 for gDNA, NM_000843.3 for mRNA, and NP_000843.2 for protein). DNA fragments from individual exons were amplified by touchdown PCR where higher annealing temperatures were set for the first five cycles, followed by moderate annealing temperature for the next five cycles and finally by a lower annealing temperature as listed in Table 1 for the remaining 23 cycles. The procedures for sequencing and variation detection were basically the same as previously described [22]. Potential mutations detected in *GRM6* of patients were further evaluated in the 96 controls by using either heteroduplex-single-strand-conformational polymorphism (HA-SSCP) [23] or polymerase chain reaction combined with restriction fragment length polymorphism (PCR-RFLP) analysis [24]. Extra pairs of primers were designed for HA-SSCP or PCR-RFLP analysis (Table 2). The 102 bp amplicons with the c.1172G>A variation were cut into 26 bp and 76 bp as the variation created a new BstNI site, while the wild amplicons remained unchanged. The 136 bp amplicons with the c.1537G>A variation could not be digested by Bsp1286I since the variation erased the Bsp1286I site that presented in wild type amplicons. The variation of c.1172G>A was genotyped and statistically analyzed using continuity correction of Pearson’s Chi-Square test with a significance level of 0.05.

**Table 1:** Primers used for polymerase chain reaction amplification and direct sequencing of *GRM6*.

**Exons**	**Primer sequence(5’-3’)**	**Product length (bp)**	**Annealing temperature (°C)**	**Note**
1	F-CCGAGCGCCTTCTCCCCAGGAC	785	68	
	R-TGCGTCGGGCTCAACTGACAGG			
2	F-TGTGCCCCGTCCGTGTCC	481	63	
	R-TGAGGCAGGGCTGAGTCGTC			
3	F-GGGCGGGACCTCTTTGTT	521	65	
	R-GCTATTCAGTCTGGGCTTGTG			
4~7	F-CCGGCCCCGTTTTTCCTG	4132	69	LA Taq DNA Polymerase
	R-CACCTGGGACGCACAAAACAC			
	F1-GCCATGCCTAGTTCACCT	/	/	Sequencing primer
	F2-ATTTGCACGTCCCTTATGAGC			Sequencing primer
	R1-CTTTCCTCGCACGCCATCC	/	/	Sequencing primer
	R2-TCAGCCTCACCCAGCCCTTCC			Sequencing primer
8	F-TCAACGAGAACGGAGATGC	900	61	
	R-CCCTTTTGGCTTTGTAACG			
9	F-CTGAGAGCCACCACGAAGAG	567	65	
	R-GCAGCCAGATACGGGATAAA			
10	F-GGGCCCCTGCTCATACTGTT	460	65	
	R-CTCCCTGCCACTGACTGTTC			

In the "Primer sequence" column, "F" indicates the forward primer and "R" indicates the reverse primer. GC1 buffer (Takara Biotechnology Dalian CO. Ltd, Liaoning, China) is designed for amplification of templates having complex secondary structure or high GC content. LA Taq DNA Polymerase (Takara Biotechnology Dalian CO. Ltd, Liaoning, China) was designed for amplification of long targets. The amplicon from exon 4 to 7 was sequenced with the additional primers listed as F1, F2, R1 and R2.

**Table 2:** Primers used for evaluating the variations by HA-SSCP or PCR-RFLP analysis of *GRM6*.

**Variations**	**Primer sequence (5’-3’)**	**Product length (bp)**	**Annealing temperature (°C)**	**Note**
c.68-82delAGGCGGGCCTGGCGCinsT	F-CCGAGCGCCTTCTCCCCAGGAC	430	71	
	R-GGTGTCCCGCGAGCAGGTG			
c.726-50g>c	F-GGGCGGGACCTCTTTGTT	214	68-66-64	Touchdown PCR
	R-CCTGGGAATCTTGATAGACTGG			
c.858-5a>g	F-GGAAGGTGCCCTAAATGC	202	65	
	R-AAGTGGCCGGTCAGGTTGG			
c.1172G>A	F-TAGGACAGGCGAGGAACG	102	70-68-66	Touchdown PCR
	R-TGGAGGGCGTGGGCAATG			
c.1537G>A	F-GGATCCCGGAGGCAGACG	136	70-68-66	Touchdown PCR
	R-CCATCTTCTTCCGCTCCC			

### Database and online tools

 Polymorphism Phenotyping (PolyPhen) [25-27] and Sorting Intolerant From Tolerant (SIFT) [28] were used to evaluate the potential pathogenicity of sequence alterations at the protein level. Automated Splice Site Analysis (ASSA) [29] and Berkeley Drosophila Genome Project (BDGP) [30] were used for predicting the alterations of splicing sites. National Center for Biotechnology Information (NCBI), Conserved Domain Database (CDD), PSORT II, WoLF PSORT [31,32], Simple Modular Architecture Research Tool (SMART) [33,34], and pTARGET [35,36] were used for analyzing structures and functions of protein domains and predicting protein topology and subcellular localization. Search Tool for the Retrieval of Interacting Genes/Proteins (STRING) was used for showing and predicting protein interactions [37].

## Results

**Figure 1:**
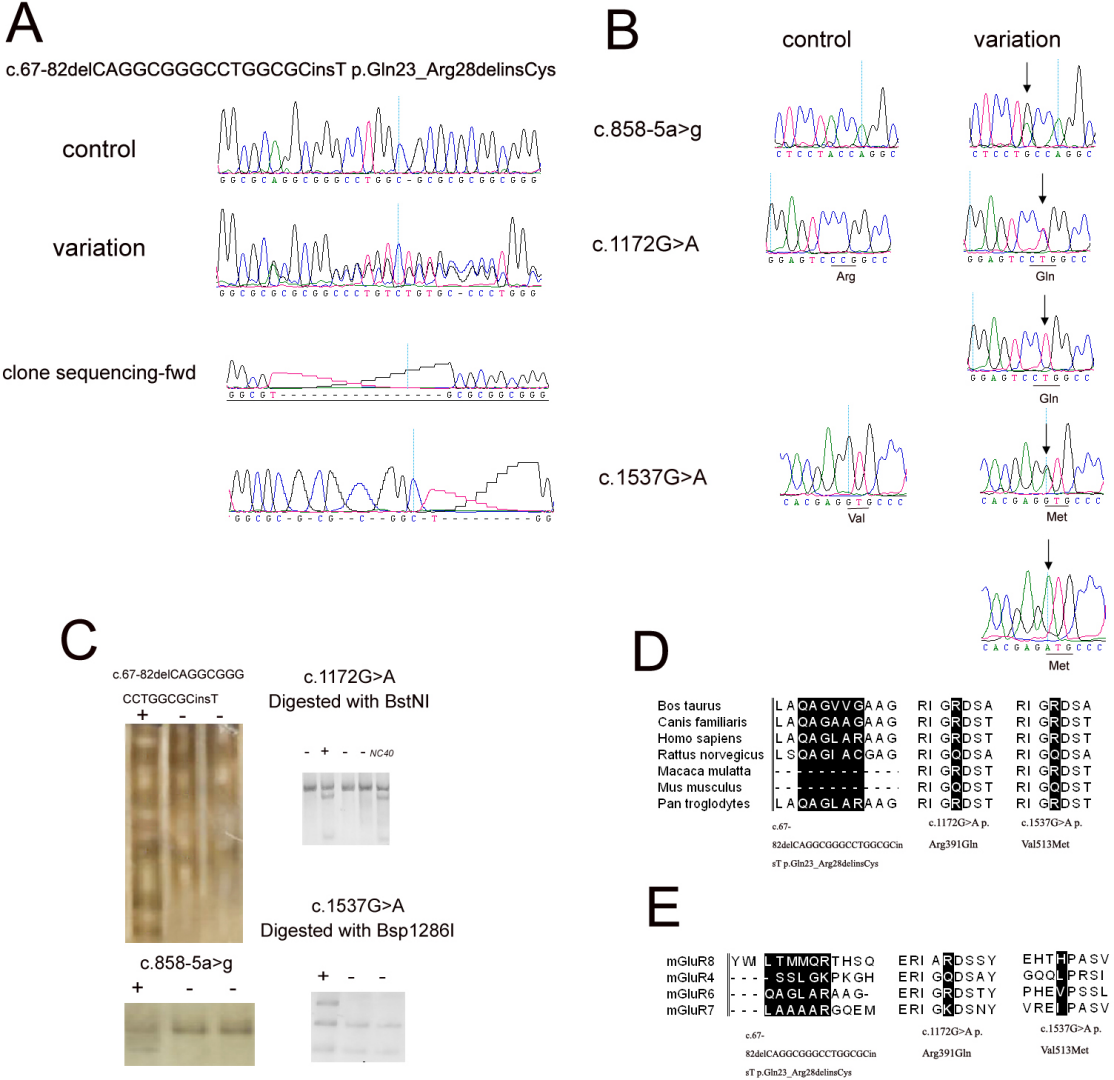
Four novel variations were detected in *GRM6* of 96 unrelated subjects with isolated high myopia. **A**: Sequences with c.67-82delCAGGCGGGCCTGGCGCinsT p.Gln23_Arg28delinsCys variation, normal control, as well as cloning sequences of both forward and reverse directions. **B**: Sequences showing the rest three novel variations as well as normal controls. The substitute bases are highlighted by an arrow, with protein consequence marked below. **C**: Variations were not observed in normal controls (NC) by SSCP or RFLP, with the exception of c.1172G>A p.Arg391Gln. Plus signs indicate samples with variation; minus signs indicate normal controls. **D** and **E**: Conservation analysis of the variations between different species (**D**) or between different members of Group III mGluRs (**E**).

Upon complete analysis of *GRM6* in 96 patients with high myopia, four novel variations predicted to have potential functional consequence were identified: c.67-82delCAGGCGGGCCTGGCGCinsT (p.Gln23_Arg28delinsCys), c.858-5a>g (r.spl?), c.1172G>A (p.Arg391Gln), and c.1537G>A (p.Val513Met; Figures 1A,B; Table 3). The c.67-82delCAGGCGGGCCTGGCGCinsT (p.Gln23_Arg28delinsCys) variation was further determined by cloning sequencing (Figure 1A). Of the four variations, three were not present in the 96 controls but the other one (c.1172G>A) was detected in four of the 96 controls. In addition, five rare variations (c.72G>A, c.504+10g>t, c.726-50g>c, c.1359C>T, and c.1383C>T) and a common variation (c.2437-6g>a) without predicted functional consequence by the abovementioned online tools [25-30] were also detected in patients with high myopia, but the presence of these variations in controls was not examined. Furthermore, nine reported SNPs (rs2645329, rs2071246, rs2645339, rs4701014, rs2067011, rs11746675, rs2071247, rs2071249, and rs17078853) were also detected in patients (Table 3).

**Table 3:** Sequence variations in GRM6 detected in 96 high myopia patients.

** **	**Variation**	**Location**	**Effect**	**High myopia (n=96)**	**Normal control (n=96)**
**#**	**c.72G>A**	**Exon 1**	**synonymous**	**1**	**ND**
**1**	**c.67-82delCAGGCGGGCCTGGCGCinsT**	**Exon 1**	**p.Gln23_Arg28delinsCys**	**1**	**0**
2	c.176A>C	Exon 1	rs2645329	76	ND
**3**	**c.504+10g>t**	**Intron 1**	**No splicing site changed**	**1**	**ND**
**4**	**c.726-50g>c**	**Intron 2**	**Splicing acceptor unaffected**	**1**	**0**
**5**	**c.858-5a>g**	**Intron 3**	**Decrease of splicing acceptor**	**1**	**0**
6	c.1131C>T	Exon 5	rs2071246	87	ND
**7**	**c.1172G>A**	**Exon 6**	**p.Arg391Gln**	**2**	4*
8	c.1227C>T	Exon 6	rs2645339	84	ND
9	c.1308T>C	Exon 6	rs4701014	96	ND
10	c.1353T>C	Exon 6	rs2067011	84	ND
**11**	**c.1359C>T**	**Exon 7**	**synonymous**	**2**	**ND**
**12**	**c.1383C>T**	**Exon 7**	**synonymous**	**1**	**ND**
13	c.1392A>G	Exon 7	rs11746675	75	ND
**14**	**c.1537G>A**	**Exon 8**	**p.Val513Met**	**3**	**0**
15	c.2196G>A	Exon 9	rs2071247	52	ND
**16**	**c.2437-6g>a**	**Intron 9**	**No splicing site changed**	**19**	**ND**
17	c.2457G>A	Exon 10	rs2071249	19	ND
18	c.2634+56a>t	Intron 10	rs17078853	19	ND

The novel variations are highlighted in bold. An asterisk indicates that there is no statistical difference between patients and controls. ND: no detected.

The c.67-82delCAGGCGGGCCTGGCGCinsT (p.Gln23_Arg28delinsCys) variation was detected in one patient in a heterozygous status, but not in the 96 controls. It was a del_ins variation without frame shifting, resulting in the deletion of six residues and the addition of cysteine at protein. Conserved domain analysis showed the affected oligopeptide was not conserved among different species or different members of Group III mGluRs (Figures 1D, E). Functional domain prediction revealed that amino acid residues one to 24 of mGluR6 would act as the signal peptide, and the following 25 to 585 amino acid residues would form an extracellular N-terminal domain related to periplasmic ligand binding. The NH_2_-terminal domain is vital in glutamate binding and the activation/inactivation of mGluR6 [38,39].

The c.858-5a>g variation was detected in one patient, but not in the 96 controls. Analysis of the corresponding splicing site by both the ASSA server and the BDGP server revealed a comparatively weak leaky effect (from 9.6 to 9.3), resulting in a –1.2 fold decrease on the natural splicing acceptor site.

The c.1172G>A variation resulted in a substitution of arginine (a strongly basic residue) to glutamine (a small polar amino acid; p.Arg391Gln), with a residue weight of one on Blosum 62 and “benign” or “tolerated” effect by PolyPhen and SIFT, respectively. The arginine in this position was conserved between different species except in *Rattus norvegicus* and *Mus musculus*, whereas it varied in different members of Group III mGluRs (Figures 1D, E). The residue with variation is in the NH_2_-terminal domain and nearby one of the key positions of the glutamate binding pocket p.Lys400 [40], presumably and, therefore, is presumed to affect the receptor family ligand binding region according to domain analyzing databases. However, this variation was found in two patients and four controls, with no statistical significance on continuity correction of Pearson’s χ^2^ test (Table 3).

The c.1537G>A variation was found in three high myopia patients, where two were heterozygous and one homozygous. It resulted in a substitution of a hydrophobic valine by a highly conserved sulfur-containing and polar methionine (p.Val513Met), with a Blosum 62 score of one and a “benign” or “tolerated” effect by the abovementioned online tools. Residues 513 to 564 were predicted to form a highly conserved extracellular domain of family 3 GPCR forming disulphide bridges (Figures 1D, E). The function of this region remains uncertain but may play a role in calcium sensing and proper trafficking of proteins [41,42]. Residue 524 is predicted to be one of the nuclear localization signals of mGluR6. Residue 513 may play a role in nuclear localization and translocation of mGluR6.

## Discussion

High myopia usually occurs alone (nonsyndromic) but, in rare cases, may present as a sign in a number of syndromes (syndromic). Genetic factors have been demonstrated to play an important role in the development of high myopia [43,44]. The genes responsible for nonsydromic high myopia are mostly unknown although identification of such genes has been sought by using various approaches, including linkage analysis, case-control association study, and sequence analyzing of candidate genes [45]. Of these, a number of studies have tested the association of nonsydromic high myopia with common SNPs in genes responsible for syndromic high myopia but, unfortunately, the results are inconclusive and controversial in general [40,46-49]. This approach is based on the understanding that the genetic basis of complex diseases is associated with common variant (common disease, common variant [CDCV]). However, routine association study of common SNPs may not discover rare variations that might contribute to complex diseases (common disease, rare variant [CDRV]) [50-52]. Sequencing the functional regions of the target gene, as in this study, would detect such rare variations as well as mutations, which should be a preferable approach for a disease like high myopia where both Mendelian traits and complex traits are involved.

Here in this study, the entire coding and adjacent intronic regions of *GRM6* were sequenced for 96 patients with nonsyndromic high myopia. Consequently, four novel variations were detected in *GRM6* of the Chinese patients with high myopia, where three were only present in patients but not among the 96 controls. These variations were predicted to affect the functions of the encoded proteins if the mutant allele is expressed. This was the first sequencing evaluation of *GRM6* in a group of patients with high myopia. The information obtained based on the current case-control sequence analyzing may not lead to a definite conclusion; however, it not only expands our understanding of *GRM6* variations in human beings but also may suggest a potential role of *GRM6* rare variations in the development of myopia in rare cases. Further studies in other ethnic populations may provide useful information to verify our findings.
